# Cisplatin's dual-effect on the circadian clock triggers proliferation and apoptosis

**DOI:** 10.1016/j.nbscr.2020.100054

**Published:** 2020-07-30

**Authors:** Zuhair Sadiq, Elizabeth Varghese, Dietrich Büsselberg

**Affiliations:** Department of Physiology and Biophysics, Weill Cornell Medicine-Qatar, Education City, Qatar Foundation, Doha, P.O. Box, 24144, Qatar

**Keywords:** Cisplatin, Cancer, Circadian clock, DNA repair, Chronotherapy, Apoptosis, Proliferation, Melatonin, CDDP

## Abstract

The circadian clock, which generates the internal daily rhythm largely mediated through release of melatonin, can be disrupted in various ways. Multiple factors result in a disruption of the circadian cycle in the clinical context, of interest are anti-cancer drugs such as cisplatin. Cisplatin modulates the circadian clock through two mechanisms: 1) the circadian clock control of DNA excision repair and 2) the effect of circadian clock disruption on apoptosis. Cisplatin can stimulate multiple classified molecules, including DNA repair factors, DNA damage recognition factors and transcription factors in drug resistance and cisplatin-induced signal transduction. These factors interact with each other and can be transformed by DNA damage. Hence, these molecular interactions are intimately involved in cell proliferation and damage-induced apoptosis. Cisplatin has a dual-effect on circadian genes: upregulation of CLOCK expression causes an increase in proliferation but upregulation of BMAL1 expression causes an increase in apoptosis. Therefore, the interference of circadian genes by cisplatin can have multiple, opposing effects on apoptosis and cell proliferation, which may have unintended pro-cancer effects. Melatonin and intracellular Ca^2+^ also have a dual-effect on cell proliferation and apoptosis and can disrupt circadian rhythms.

## Highlights

• Cisplatin has a dual-effect on components of the circadian clock, increasing or decreasing cell proliferation and apoptosis.
• DNA excision repair and apoptosis are controlled by circadian rhythms.
• When cisplatin is combined with other agents, the effects are enhanced.
• These findings provide clinicians with the prospect to create effective chrono-cisplatin regimens for patients.

## Abbreviations

BAX: bcl-2-associated X protein
BMAL1: brain and muscle ARNT-like 1
CaM: calmodulin signaling pathway
CDDP: cisplatin
CLOCK: clock circadian regulator
CRY: crystal
DNA: deoxyribonucleic acid
EMT6: mammary carcinoma cell line
IP_3_: inositol trisphosphate
LLC: lewis lung carcinoma cell line
NER: nucleotide excision repair
PER: period
RNA: ribonucleic acid
SCN: suprachiasmatic nucleus
UV: ultraviolet
XPA: xeroderma pigmentosum group A

## 1 Introduction

Cisplatin (CDDP) is a chemotherapy medication used to treat numerous solid tissue cancers such as testicular, ovarian, lung and breast cancers (Dasari and Tchounwou, 2014). While it has several actions (Florea and Büsselberg, 2011), cisplatin's primary mechanism of actions is through DNA damage inside cancer cells, thereby preventing them from multiplying. However, healthy cells are also affected, initiating serious side effects that may cause physicians to stop the treatment (Shirmanova et al., 2017). Both healthy and cancerous cells partially repair the DNA damage caused by anti-cancer drugs. Successful cancer treatment requires killing cancerous cells that have DNA damage when they are least capable of repairing it, while not destroying the healthy cells (Kaliberov and Buchsbaum, 2012). Several molecular mechanisms leading to cell death are associated with the use of cisplatin treatment in chemotherapy. Cisplatin can induce apoptosis by triggering signal transduction pathways and directly interacting with DNA, mitochondria and calcium channels (Kohno et al., 2015).

Circadian rhythms are produced by the body's biological clock. The center is situated in the suprachiasmatic nucleus (SCN) in the hypothalamus that contains molecular machinery that produces circadian rhythms (Gillette and Tischkau, 1999; Richter et al., 2004). The disruption is linked to an increased incidence of cancer, obesity, diabetes, cognitive problems, and cardiovascular disease (Shanmugam et al., 2013). Abnormal exposure to light during the dark phase of the circadian clock disrupts the activity of the suprachiasmatic nucleus and causes anomalous effects to downstream processes which can cause cancers (Subramanian et al., 2010). The circadian system modulates physiological parameters such as cell-cycle control, proliferation, apoptosis and DNA damage repair (Liu et al., 2015). Mainly two epigenetic processes are linked to circadian clock. 1) DNA methylation which regulates core clock gene expression and the circadian clock and 2) miRNAs fluctuate with circadian rhythms and effect underlying mechanisms of the circadian clock (Masri et al., 2013; Joska et al., 2014).

A major player in generating the circadian rhythm is melatonin, a serotonin-derived hormone. Melatonin is produced by various tissues in the human body; the major source is the pineal gland, which is a small endocrine gland in the brain of most vertebrates. Although the exact mechanisms of action are still under discussion, current evidence suggests that secretion is controlled by circadian genes and night/day cycles (Reppert and Weaver, 1995). Clinical studies suggest that melatonin decreases sleep latency and improves sleep duration. Melatonin is also given to patients undergoing chemotherapy. It consistently enhances the effects of chemotherapy while reducing some side effects, like weight loss and nerve pain. *In vitro* and *in vivo* studies show that melatonin has antioxidant and antiproliferative properties and synergistic effects with anti-cancer agents (Reiter et al., 2001).

Clock-dependent processes influence the success of anti-cancer treatment (Table 1, Table 2). Chronotherapy describes how the effectiveness of anti-cancer drugs such as cisplatin changes on the time of administration and, therefore, on the internal clock (Eriguchi et al., 2003). Timing of cisplatin's administration that targets cancer cells actively replicating their DNA improves the effectiveness of the treatment while reducing healthy cell death (Li et al., 2015).

**Table 1:** Anti-cancer drugs with known significance of circadian timing for antitumor efficacy in laboratory mice. Circadian timing linked with highest tolerability in mice in hours after light onset. The demonstration of chrono-efficacy rests on the administration of a single anticancer drug over a span of days and weeks. Appropriately circadian-timed chemotherapy significantly decreases tumorigenesis and increases lifespan in mice with tumors.

Class of Drug	Name	Circadian timing associated with best tolerability in mice (hours after light onset, 0–24)	Reference
Antimetabolite	5-fluorouracil	7	Terazono et al. (2008)
	L-alanosine	19.5	Li et al. (2006)
Intercalators	Theprubicin	10	Lévi et al., 1988a, Lévi et al., 1988b
Alkylators	Cisplatin	14	Dakup et al. (2018)
	Oxaliplatin	15	Lévi et al. (2000)
	Carboplatin	15	Ron et al. (1998)
Mitosis inhibitor	Vinorelbine	19	Li et al. (2002)

**Table 2:** Expression of circadian genes differs in various forms of cancers. Circadian genes have a critical role in the cell cycle and aberrant expression are implicated in cancerogenesis.

Cancer Type	Circadian Gene	Reference
Prostate Cancer	*BMAL1, CLOCK, CRY1, CRY2, PER1, PER2, PER3*	Jung-Hynes et al. (2010)
Colon Cancer	*PER2*	Wood et al. (2009)
Breast Cancer	*CLOCK, CRY1, CRY2, PER1, PER2, PER3*	Sahar & Sassone-Corsi (2007)
Lung Cancer	*CLOCK, PER1, PER2, PER3*	Gery et al. (2007)
Non–Hodgkin Lymphoma	*BMAL1*	Taniguchi et al. (2009)
Glioma	*CRY1, CRY2, PER1, PER2, PER3*	Fujioka et al. (2006)

**Fig. 1:**
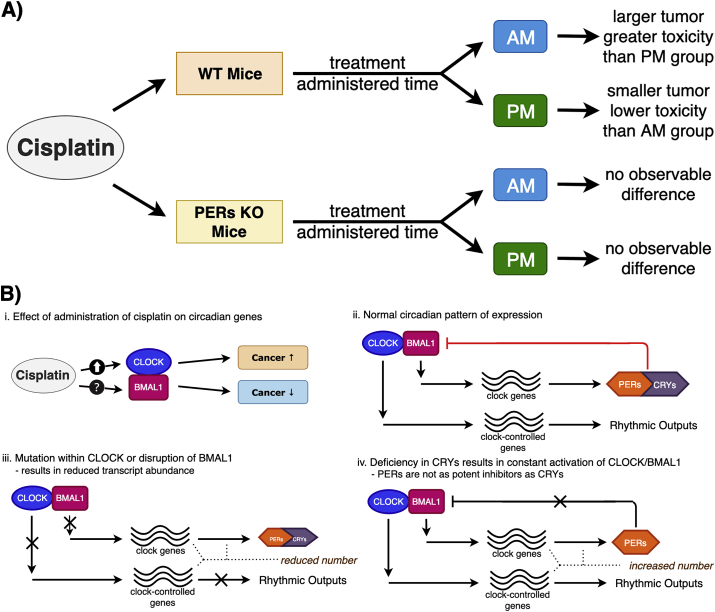
**A)** Circadian rhythms regulate tumor regression and cisplatin-induced toxicity. Murine models have revealed that the time cisplatin is administered during the day influences renal and blood toxicities. Cisplatin chrono-pharmacology encompasses the circadian cycle control of DNA repair. **B)** The core transcription-translation feedback loop in the circadian system consists of a primary loop of CLOCK-BMAL1 and PER-CRY complexes. CLOCK and BMAL1 triggers the expression of PER, CRY, clock gene and clock-controlled genes. Mutations within CLOCK/BMAL1 or disruption of the feedback loop can lead to carcinogenic effects. Cisplatin has a dual, opposite effect on CLOCK and BMAL1.

In murine studies, cisplatin's effectiveness was increased, and its toxic side effects were decreased when the drug was administered at certain times of the day. Mice had less nephrotoxic effect sand overall better kidney function tests when they received cisplatin when their urine output was highest versus mice who received cisplatin when their urine output was lowest (Lévi et al., 1988a, Lévi et al., 1988b) (Fig. 1a). Cisplatin often has adverse side effects on the kidney; therefore, these results might indicate how to reduce side effects.

Because mice are nocturnal animals, the circadian clock changes differ from those of humans (roughly a 12hr phase shift) (Mure et al., 2018). Chronotherapy gets further complicated in patients who are taking multiple drugs. However, recent studies have shown that chronotherapy can be beneficial to cancer patients undergoing combination therapies (Selfridge et al., 2016). For example, patients with ovarian, endometrial and bladder cancer that received doxorubicin or pirarubicin at a certain time in the morning and cisplatin 12 h later had decreased toxicity and greater tumor response and survival than patients who had doxorubicin or pirarubicin in the evening and cisplatin 12 h later (Kobayashi et al., 2009).

However, there is limited knowledge how cisplatin influences the circadian clock (Fig. 1b). Here we compile information on the molecular connection how cisplatin modulates the circadian clock and their overlapping pathways will be discussed. Since various studies have found subjects respond differently to chemotherapeutic drugs compared to wild-type controls, the results suggest cisplatin interferes with and changes the circadian clock. The interference of circadian genes by cisplatin have opposing carcinogenic effects. We found evidence that cisplatin modulates the circadian clock through two approaches-one approach is centered on the circadian rhythm control of DNA excision repair and the other approach is centered on the effect of circadian cycle interference on apoptosis.

## 2 Cisplatin modulates the circadian clock by circadian genes, melatonin and [Ca^2+^]_i_

Alkylating agents like cisplatin work through various different mechanisms that accomplish the same result, which is the interference of DNA function and apoptosis. 1) Addition of alkyl groups to DNA bases results in DNA being split by repair enzymes as they try to substitute the alkylated bases, blocking DNA synthesis and RNA transcription from the affected DNA, 2) DNA damage due to the formation of cross-links, which are bonds between atoms in the DNA, that prevents the strands from being detached for transcription or synthesis, and 3) the induction of nucleotide mispairing, which can lead to mutations (Dasari and Tchounwou, 2014). In addition, cisplatin induces reactive oxygen species, which triggers apoptosis alongside DNA damage (Brozovic et al., 2010). Several other molecular mechanisms of action include induction of cell cycle arrest and p53 signaling, increase in intracellular calcium, triggering of both extrinsic and intrinsic pathways of apoptosis, and down-regulation of proto-oncogenes and anti-apoptotic proteins (Florea and Büsselberg, 2011).

### 2.1 Cisplatin has a dual-effect on circadian genes: upregulation of CLOCK expression causes an increase in proliferation while upregulation of BMAL1 expression causes an increase in apoptosis

Only a few studies have studied the association between circadian genes and cisplatin. A study verifying the link between the CLOCK gene and cisplatin resistance treated cisplatin-resistant and cisplatin-sensitive ovarian cancer cells with varying concentrations of cisplatin. CLOCK protein expression amplified as cisplatin concentration increased in the cisplatin-resistant and cisplatin-sensitive cell lines in a dose-dependent way, suggesting that the CLOCK gene and protein were linked with cisplatin resistance in ovarian cancer cells causing an increase in cancerogenesis (Xu et al., 2018).

**Fig. 2:**
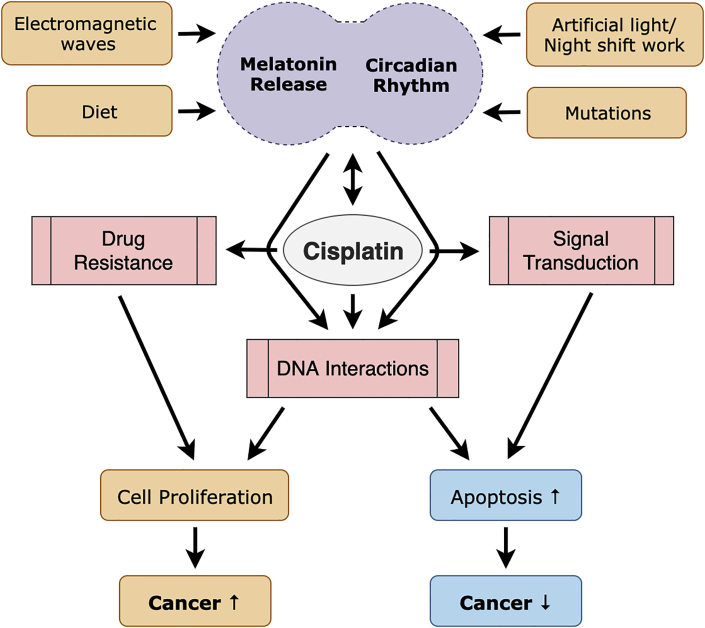
Circadian cycles are disrupted in various ways. Numerous environmental factors, such as night shift work, diet, and exposure to electromagnetic waves and artificial light, result in circadian disruption mostly by changing melatonin rhythms. The circadian clock is also mediated by anti-cancer drugs including cisplatin. Cisplatin affects drug resistance, DNA interactions and signal transduction and has various mechanisms and targets. Cisplatin activates signal transduction pathways and directly interacts with DNA, which can induce apoptosis or cell proliferation. Cisplatin can stimulate numerous categorized molecules, including DNA repair factors, DNA damage-recognition factors and transcription factors involved in drug resistance and cisplatin-induced signal transduction. These factors interact with each other and may be transformed by DNA damage. Hence, these molecular interactions are meticulously involved in cell proliferation and damage-induced cell death.

In order to comprehend how the BMAL1 gene affects tumor growth and its response to cisplatin, a study examined the effect of knockdown of BMAL1 by RNAi. Downregulation of BMAL1 gene expression enhanced cell growth *in vitro* and promoted tumor growth in mice. Inhibiting BMAL1 expression in the mice's fibroblast cells and colon cancer cells decreased DNA damage caused by cisplatin. Knockdown of BMAL1 decreased the expression of PER1, PER2, PER3, and p53. BMAL1 contributes to regulating cell-cycle progression, tumor cell apoptosis, and DNA-damage response and in homeostasis regulation by accelerating the development of tumors and influencing the response to cisplatin (Zeng et al., 2010). These outcomes demonstrate that the interference of clock genes (CLOCK and BMAL1) can have opposite oncogenic effects (Fig. 2).

### 2.2 Melatonin reduces cisplatin-initiated effect on apoptosis and proliferation

Melatonin regulates the sleep-wake cycle. It inhibits human cancer cell proliferation *in vitro* and new research confirms its anti-cancer properties *in vivo*; *in vitro* studies demonstrate that melatonin has antiproliferative effects on breast cancer (Blask, 2009). Melatonin is an effective free-radical scavenger and enhances antioxidative enzyme activities, interacting with cytosolic calmodulin (a calcium-binding protein) and inducing apoptosis (Sainz et al., 2003). Melatonin regulates the Ca^2+^ and the calmodulin-signaling pathway (CaM) either by varying [Ca^2+^]_i_ via a direct interaction with CaM or through activation of its G-protein coupled membrane receptors (Dai et al., 2002). Melatonin may exert protective effects against calcium overload in the cytoplasm and cell damage by reducing Ca^2+^ release via the stimulation of Ca^2+^ transport from cells through the membrane transporters and consequent Ca^2+^ re-uptake into the endoplasmic reticulum (del Castillo-Vaquero et al., 2010; Santofimia-Castaño et al., 2014). However, in mice models, melatonin increases [Ca^2+^]_i_ in the liver, muscle, white adipose tissues and pancreas (Agil et al., 2015). As such, results from studies on the effect of melatonin of [Ca^2+^]_i_ are contradictory.

**Fig. 3:**
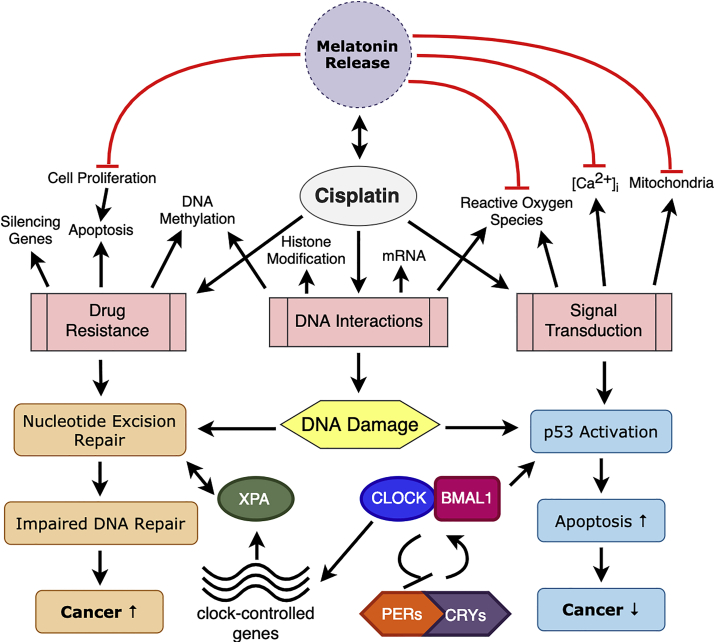
Model for DNA damage and its regulation by circadian rhythms after treatment with cisplatin. Melatonin decreases cisplatin-induced toxicity by promoting apoptosis, reducing [Ca^2+^]_i_ and mitigating molecular damage associated with ROS generation in the mitochondria. Melatonin acts as a therapeutic option for preventing the toxic effects of cisplatin, because of its proven ability to be a direct free radical scavenger, indirect antioxidant and natural programmed cell death inducer in cancer. One mechanism how cisplatin kills cancer cells is by fastening to DNA and impeding with its repair mechanism, ultimately causing apoptosis. However, it can have an unintended effect that ultimately decreases apoptosis. Both pathways are regulated by circadian clock. Cisplatin's cytotoxicity occurs due to a failure of the nucleotide excision repair (NER) system. During NER, the damaged parts of DNA has to be recognized by the cell before it is removed from the rest of the strand. The cell detects DNA damage by the action of damage recognition proteins and results in DNA repair instead of apoptosis, having a carcinogenic effect on the cell.

Melatonin enhances the anti-tumor effect when used in conjunction with anti-cancer drugs (Fig. 3). This synergism is due to the increase in apoptosis and the substantial increase in the triggering of both extrinsic and intrinsic apoptotic pathways when melatonin is administered with chemotherapy (Casado-Zapico et al., 2010). Concurrent administration of melatonin with cisplatin considerably decreases the incidence of neurotoxicity, cardiotoxicity, and respiratory weakness for lung cancer patients (Lissoni et al., 1999; Song et al., 2012).

Oxidative stress produced by free radicals contributes to the increase of cisplatin-induced testicular damage. Melatonin has a strong protective effect against testicular toxicity caused by cisplatin (Ilbey et al., 2009). It was immuno-cytochemically shown that melatonin reduced the fluorescent-signal linked to reactive oxygen species (ROS) generation in the mitochondria (Reiter et al., 2018). Melatonin increased cisplatin-induced apoptosis by modifying cytochrome c and caspase signaling pathway (Hao et al., 2017). Hence, melatonin could act as a possible therapeutic agent for the impairment of cellular function, which is caused by cytotoxic anti-cancer drugs such as cisplatin.

### 2.3 Cisplatin induced increase of [Ca^2+^]_i_ has a dual-effect on cells and can disrupt the circadian clock

Intracellular calcium signaling has a key role in controlling when and how genes are turned off and on in response to environmental stimuli in the majority of organ systems. Hence, calcium plays a fundamental role in regulatory networks underlying the circadian clock, and as such, the biological clock in every cell. For example, quiescent human cell lines such as human bone osteosarcoma epithelial cells have robust 24-h calcium rhythms. In the SCN, the circadian cycles of [Ca^2+^]_i_ are observed at the level of individual neurons and the cellular network (Splettstoesser et al., 2007; Ikeda et al., 2003). Blocking calcium influx stops the periodic expression of circadian genes in the SCN (Lundkvist et al., 2005). In addition, BMAL1 is critical for circadian calcium rhythms. Inhibition of BMAL1 expression causes period elongation and amplitude reduction in calcium rhythms of SCN cells (Ikeda and Ikeda, 2014).

Calcium channels and pumps are involved in many cancers (Monteith et al., 2012). Dysregulated calcium homeostasis contributes to tumorigenesis. As such, various anti-cancer drugs including cisplatin target calcium signaling proteins and, typically, increase [Ca^2+^]_i_ inducing apoptosis in tumor cells. [Ca^2+^]_i_ can be modulated by active and passive transport on the cell membrane. Cisplatin can increase [Ca^2+^]_i_ through many mechanisms. One mechanism for how cisplatin increases [Ca^2+^]_i_ concentration is by opening up a calcium pore on the cell membrane or an IP_3_ receptor, which causes a calcium influx into the cells from the extracellular space. Other mechanisms as to how anti-cancer drugs increase [Ca^2+^]_i_ through the release of calcium from the intracellular stores and inhibition of active calcium transporters, which prevent the movement of calcium from the intracellular space to the extracellular space (Florea and Büsselberg, 2009). While increasing [Ca^2+^]_i_ generally leads to a reduction of cancer, it also has negative side-effects on cells. Calcium can kill healthy cells and cause renal toxicity, neurotoxicity and autoimmunity (Florea and Büsselberg, 2006).

## 3 Cisplatin modulates DNA repair via circadian genes that causes either upregulation or downregulation of cell proliferation

Cisplatin produces covalent bonds crosslinking DNA bases in an inter-strand or intra-strand manner (Guainazzi and Schärer, 2010). The covalent crosslinks prevent DNA replication and mitosis and, in turn, causes a DNA repair response. If the cell cannot remove the crosslink, it is either apoptized or, if apoptosis is repressed, cannot make viable daughter cells. Nevertheless, tumorigenesis is repressed.

The circadian clock interfaces at various points with the cellular response to DNA damage (Fig. 3). As such, the clock plays a significant role in cancers induced by chemical agents, which damage the genetic information within a nucleus producing mutations, in addition to playing a role in treating cancers by DNA damaging agents (Damulewicz et al., 2019). Nucleotide excision repair (NER) is critical for preserving genomic integrity as it is the only system that can repair a wide array of DNA damage (Garfinkel and Bailis, 2002). NER is currently the only understood means through which bulky adducts including those generated by platinum-based chemotherapies such as cisplatin, are detached from DNA in human cells leading to DNA repair and decrease in the effectiveness of the anti-cancer drug (Kang et al., 2009).

Apart from the regulatory association between DNA damage repair and the circadian clock, NER has a circadian rhythm in mice, hypothesized to be because of fluctuations in XPA protein expression, the DNA damage recognition protein for this pathway (Gaddameedhi et al., 2011). XPA protein has a vital role in cisplatin damage repair by NER and demonstrates circadian oscillation in the liver of mice (Kang et al., 2010). The circadian oscillation of XPA is attained via regulatory mechanisms of transcription dictated by core circadian clock proteins such as cryptochrome. NER is continually elevated in CRY-deficient mice, suggesting that circadian rhythms downregulate the activity of NER during particular times of the day (Kang and Leem, 2014).

Analysis of NER activity during the span of a day in mice tissue showed that the repair activity has circadian cycles. By damaging DNA with cisplatin and monitoring NER over an entire circadian cycle, Yang et al. (2018) obtained circadian repair maps for mouse kidney and liver. Their quantitative analyses determined which strand of which gene is repaired at a given time of the day for the entire mouse genome and revealed that for many genes the transcribed strand and non-transcribed strand are repaired out of phase (Yang et al., 2018). Similar experiments in mice showed that transcription-driven repair is nearly complete after 2 days post-DNA damage, whereas after several weeks for repair of the non-transcribed strand and the rest of the genome (Yang et al., 2019). Transcribed strand repair oscillates in rhythmically expressed genes up to 2 days post-DNA damage, and in all expressed genes, there was an increase in transcribed strand repair with time from the 5′ to 3′ end (Yang et al., 2019).

Furthermore, transcriptional regulation of core clock proteins affects the oscillation of the XPA protein. Since XPA plays a role the initial phase of NER and is the rate-limiting factor, a time-dependent discrepancy in its relative abundance caused a diminished DNA repair ability when UV exposure happened out of phase with its expression (Gaddameedhi et al., 2011). DNA repair efficiency and damage induction are both specifically affected by the phase of circadian cycles during which the cells are exposed to UV (Sancar et al., 2010). The results attained by suppressing BMAL1 expression showed that the circadian cycles play a critical role in adjusting DNA sensitivity to UV light and the subsequent DNA repair process (Sancar et al., 2015).

Researchers found a direct link between circadian rhythms and p53 via the clock-intrinsic apoptosis pathway. p53 lacks robust circadian cycles, however it appears that BMAL1 upregulates p53 expression (Mullenders et al., 2009). Furthermore, knockdown of p53 seems to diminish the amplitude of circadian rhythms in cell-based assays, while p53 mutant mice have an apparently normal circadian clock (Zhang et al., 2009).

## 4 Cisplatin disrupts apoptosis through the activation of pathways that are parallel to the expression of circadian genes

The goal of cisplatin is to preferentially kill cancer cells relative to healthy cells. A main mechanism of cisplatin-induced cell killing is apoptosis. Cisplatin can trigger several parallel pathways that lead to cell cycle arrest and apoptosis; this is dependent on treatment conditions, cellular environment, cell type or cisplatin concentration (Köberle et al., 2010). The most well understood pathway of cisplatin is the activation of DNA damage signaling pathways which trigger mitochondrial apoptosis (Siddik, 2003). p53 is also implicated in the activation of apoptosis caused by DNA damage by cisplatin. Additionally, p53 is a transcriptional activator and, as such, increases the transcription of several genes. The pattern of transcriptional regulation is important to determine a cell's response to DNA damage (Lowe et al., 1994). p53 protein specifically binds to DNA sequences to stimulate growth of inhibitory genes or non-specifically to damaged sites leading to DNA repair or apoptosis. Loss of binding of p53 protein to damaged DNA causes a failure of cells to prompt apoptosis and can cause resistance to anti-cancer drugs (Liu and Kulesz-Martin, 2001).

In contrast to p53, the proto-oncogene *c-myc* stimulates either apoptosis or cell-cycle progression; *c-myc* expression levels closely relate to cell proliferation (Leon et al., 2009). The expression of *wee1* and PERs is controlled by the circadian variation of the complex CLOCK-BMAL1. PER genes act as tumor suppressors. Overexpression of PER1 made human cancer cells susceptible to DNA damage-induced apoptosis; conversely, inhibition of PER1 in the same cells decreased cell death (Gery et al., 2006).

Overexpression of mPER2 in the Lewis lung carcinoma cell line of mice and mammary carcinoma cell line resulted in decreased cell proliferation and increased apoptosis (Hua et al., 2006). Overexpression of mPER2 also changed the expression of apoptosis-related genes. The mRNA and protein levels of c-Myc, Bcl-XL and Bcl-2 were decreased, while the expression of p53 and BAX was increased in mPER2-overexpressing cells. Furthermore, overexpression of PER1 resulted in apoptosis and inhibition of growth in prostate cancer cells. To recognize the effect of CRY on cancer, mice models that had CRY knocked out were merged with a p53-null mutation. Those mice developed tumors (Ozturk et al., 2009).

### 4.1 Cisplatin can increase the risk of developing leukemia

As mentioned earlier, cisplatin is involved in the treatment of numerous cancers including ovarian, lung, testicular, and bladder cancers. It works against various types of cancers, including lymphomas, sarcomas, carcinomas, and germ cell tumors (Dasari and Tchounwou, 2014) (Table 3). However, cisplatin increases the risk of leukemia. The risk of leukemia rises when the concentration increases. The risk of developing leukemia increases more if radiation is given along with cisplatin (Travis et al., 1999).

**Table 3:** Cisplatin has a dual-effect on cancerogenesis, which hinges on the type of cancer and the circadian genes involved.

Cancer Type	Circadian Gene	Cancer	Reference
Leukemia	*BMAL1*	Increase	Rahman et al. (2017)
Breast Cancer	*CLOCK, CRY1, CRY2, PER1, PER2, PER3*	Decrease	Lesicka et al. (2018)
Lung Cancer	*CLOCK, PER1, PER2, PER3*	Decrease	Liu et al. (2014)
Ovarian Cancer	*CLOCK*	Decrease	Xu et al. (2018)

## 5 Discussion

Cisplatin-focused chronotherapy has an advantage in alleviating side effects of anti-cancer drugs, and cisplatin can influence the circadian clock (Dakup et al., 2018). Past results present exciting outlooks for chemotherapy, in addition to other treatments that focus on DNA damage such as radiation therapy. However, the chronotherapeutic use of cisplatin is in a delicate balance between augmenting the treatment of cancer and reducing unintended side effects. It is critical to recognize the mechanisms that affect chronotherapeutic outcomes to find this right balance. Cisplatin can modulate the circadian clock causing an increase in cell proliferation. It is worth noting that the effect of anti-cancer drugs on specific circadian genes is a novel area of exploration in chronotherapy research and more experimental data is necessary for a more definitive conclusion. Nonetheless, the considerable value that cisplatin offers patients with advanced cancer offsets the comparatively smaller risk of cell proliferation and cancerogenesis.

Melatonin attenuates cisplatin-induced damage in cells. Melatonin is a potent antioxidant and protects cells against oxidative stress triggered by DNA damage and ROS. Studies show that smaller melatonin dosages (5 or 10 mg/kg) are inadequate to guard the cells from cisplatin-induced toxic effects. However, when higher doses (20 mg/day) were used alongside anticancer drugs of different malignances (gastrointestinal tract, cervix, prostate, testis, and lung cancer), melatonin improved the rate of tumor regression and increased survival and quality of life of patients (Lissoni, 2002). However, future research should address if pre-treatment alongside melatonin does not impede with the circadian clock and the efficacy of cancer treatment.

Even though cancer-focused chronotherapy has numerous problems, both practical and conceptual, the need for improving therapeutic effectiveness while reducing side effects via chronobiology is strong. In order for this to become embedded in the treatment framework, it will be critical to understand which drugs are and which drugs are not affected by the circadian clock, and how the time of administration might need to be altered in a patient- and cancer-specific fashion. Overall, it is pretty clear that there is an unmet need for better tailored and effective therapeutic approaches for the treatment of cancers.

## 6 Conclusion

The recent findings that NER and apoptosis are controlled by circadian rhythms has provided clinicians with the prospect to create effective chrono-cisplatin regimens for patients. However, other aspects need to be integrated into the general approach of treating disease in a clinical setting to have consistent, definitive results.

Anti-cancer drugs like cisplatin affect components of the circadian clock causing either an increase or decrease in apoptosis and cell proliferation. When these drugs are combined with each other or with other types of agents, they can have powerful anti-cancer effects. However, more work needs to be done before a conclusion can be drawn.

More investigation of the underlying mechanisms behind chronotherapy, the effects of such responses to human physiology, the effect of tumorigenesis on the circadian cycles and the ability of the circadian clock to respond to chemotherapy are needed and would contribute greatly to advance anti-cancer chronotherapy.

## Author contributions

The authors contributed as follows: Conceptualization, Z.S. and D.B.; Writing—Original Draft Preparation, Z.S.; Writing—Review and Editing, Z.S., E.V. and D.B.; Figure Preparation and Editing, Z.S.; Visualization, Z.S. and D.B.; Supervision, D.B.; Funding Acquisition, D.B. All authors reviewed the results and approved the final version of the manuscript.

## Declaration of competing interest

We declare to have no conflict of interest for the above mentioned manuscript.
